# PI3K/AKT/mTOR Pathway-Associated Genes Reveal a Putative Prognostic Signature Correlated with Immune Infiltration in Hepatocellular Carcinoma

**DOI:** 10.1155/2022/7545666

**Published:** 2022-05-09

**Authors:** Zhihuai Wang, Adeel Ur Rehman, Xihu Qin, Chunfu Zhu, Siyuan Wu

**Affiliations:** ^1^Graduate School of Dalian Medical University, Dalian Medical University, Dalian 116044, China; ^2^Department of Hepatobiliary Surgery, The Affiliated Changzhou No. 2 People's Hospital of Nanjing Medical University, Changzhou 213000, China; ^3^Center of Gastrointestinal Disease, The Affiliated Changzhou No. 2 People's Hospital of Nanjing Medical University, Changzhou 213000, China

## Abstract

**Background:**

The dysregulated PI3K/AKT/mTOR pathway acts as the main regulator of tumorigenesis in hepatocellular carcinoma (HCC).

**Aim:**

Here, we identify the prognostic significance of PI3K/AKT/mTOR pathway-associated genes (PAGs) as well as their putative signature based on PAGs in an HCC patient's cohort.

**Methods:**

The transcriptomic data and clinical feature sets were queried to extract the putative prognostic signature.

**Results:**

We identified nine PAGs with different expressions. GO and KEGG indicated that these differentially expressed genes were associated with various carcinogenic pathways. Based on the signature-computed median risk score, we categorized the patients into groups of low risk and high risk. The survival time for the low-risk group is longer than that of the high-risk group in Kaplan-Meier (KM) curves. The prognostic value of risk score (ROC = 0.736) of receiver operating characteristic (ROC) curves performed better in comparison to that of other clinicopathological features. In both the GEO database and ICGC database, these outcomes were verified. The predictions of the overall survival rates in HCC patients of 1 year, 3 years, and 5 years can be obtained separately from the nomogram. The risk score was associated with the immune infiltrations of CD8 T cells, activated CD4 memory T cells, and follicular helper T cells, and the expression of immune checkpoints (PD-1, TIGIT, TIM-3, BTLA, LAG-3, and CTLA4) was positively relevant to the risk score. The sensitivity to several chemotherapeutic drugs can also be revealed by the signature. CDK1, PITX2, PRKAA2, and SFN were all upregulated in the tumor tissue of clinical samples.

**Conclusion:**

A putative and differential dataset-validated prognostic signature on the basis of integrated bioinformatic analysis was established in our study, providing the immunotherapeutic targets as well as the personalized treatment in HCC with neoteric insight.

## 1. Introduction

In light of the Global Cancer Statistics of 2020, hepatocellular carcinoma (HCC) was the third leading malignancy associated with deaths from cancer worldwide [1]. HCC is deemed to be the commonest type of primary liver cancer, and the 5-year survival rates decline to 14.1% in China [2]. Surgical resection is considered the optimal option for patients at the early stages, but it is still accompanied by a high risk of recurrence [3, 4]. Hence, identifying prognostic biomarkers as well as therapeutic assessment models is essential.

The phosphatidylinositol 3-kinases (PI3Ks)/protein kinase B (AKT)/mammalian target of rapamycin (mTOR) pathway was a classical intracellular signaling receptor to react extracellular stimulators. The hyperactivation of the PI3K/AKT/mTOR pathway is involved in diverse human tumors [5]. Particularly, as the HCC developed and progressed, the PI3K/AKT/mTOR pathway was dysregulated [6]. For example, the proliferation of HCC can be promoted by NCSTN through the PI3K/AKT pathway [7]. SPAG5 promoted the progression of HCC via the PI3K/AKT pathway [8], and even the participation of lncRNA FER1L4 in the PI3K/AKT pathway can give rise to the development of HCC cells [9]. Focusing on the PI3K/AKT/mTOR signaling pathway is an opportunity for HCC therapy. Therefore, it is innovative and feasible to identify the prognostic significance of PI3K/AKT/mTOR pathway-related genes (PAGs) as well as their putative signature based on PAGs in HCC.

Rapid progress had been achieved in the therapeutic strategy of liver cancer, especially the immunotherapy trend to be the hot spot among cutting-edge treatments [10, 11]. Tumor cells were inhibited and killed by mediating specific immune responses, thereby reducing the risk of HCC metastasis and recurrence [12]. Immunotherapy had been acknowledged as a valid therapeutic option for advanced liver cancer [13], but a large percentage of HCC patients had poor therapeutic efficacy in immunotherapy. Some studies indicated that the tumor microenvironment (TME) had the potential to be a prognostic indicator; it will contribute to predicting the immunotherapeutic efficacy and benefiting from precision treatments [14]. At the same time, tumor-infiltrating lymphocytes (TILs) were recognized as the indispensable component of TME [15]. Differential types of lymphocytes may be able to promote the progression of HCC; determining the infiltrative capacity of various immune cell subtypes in TME provided novel insight into immunotherapy [16]. The PI3K/AKT/mTOR which was an activated aberrant pathway can reduce the patient's response to immunotherapy by mediating the immune tolerance in TME [17]. Therefore, we further explored the correlation between the PI3K/AKT/mTOR pathway-associated prognostic signature (PAPS) and the related ratio of infiltrating immune cells in TME, aimed at monitoring the individualized immunotherapy and predicting the prognosis for HCC patients.

Current research had proposed quite a bit of prognostic signature for HCC patients [18–20], but our research developed a comprehensive and reliable prognostic signature based on PAGs for the first time, which may be capable of simultaneously predicting prognosis and immunotherapeutic efficacy in HCC. In particular, the expression of prognostic PAGs was validated by way of utilizing our clinical samples. We concluded that the signature may reveal the status of TME and direct the individualized immunotherapy in HCC; it has the potential to be a robust prognostic model and provides the HCC patients with novel insight regarding the new immunotherapy targets.

## 2. Materials and Methods

### 2.1. Collect Clinical Specimens

Under the approval of the ethical committee of the Affiliated Changzhou No. 2 People's Hospital of Nanjing Medical University, all the HCC tissues as well as corresponding normal tissues were acquired from 35 patients treated in Affiliated Changzhou No. 2 People's Hospital of Nanjing Medical University (Jiangsu, China) in 2019-2021. Each patient enrolled in the study had informed consent before collecting samples, and the HCC tissue and corresponding normal tissue were obtained from the course of surgery and were immediately stored in -80°C liquid nitrogen.

### 2.2. Real-Time Reverse Transcription-PCR

The 35 pairs of HCC tissue and corresponding normal tissue were adopted to extract total RNA; cDNA was obtained via HiScript II Q RT SuperMix for qPCR (+gDNA wiper) (Vazyme, Nanjing, China). Ultimately, the real-time PCR system (ABI, Waltham, MA, USA) was employed to proceed with the q-PCR. We bought the primers for qRT-PCR from RiboBio (Guangzhou, China). The base sequence of SFN was exhibited as forward: TCTCTCTGGCCAAGACCACT, reverse: TGATGAGGGTGCTGTCTTTG; PRKAA2: forward: GTGAAGATCGGACACTACGTG, reverse: CTGCCACTTTATGGCCTGTTA; PITX2: forward: CGGCAGCGGACTCACTTTA, reverse: GTTGGTCCACACAGCGATTT; CDK1: forward: TTTTCAGAGCTTTGGGCACT, reverse: AGGCTTCCTGGTTTCCATT; and GAPDH: forward: TGAAGGTCGGAGTCAACGGATTTGGT, reverse: CATGTGGGCCATGAGGTCCACCAC. GAPDH was considered the internal control. The formula for fold change is set as fold change = 2^−ΔΔCT^.

### 2.3. Obtaining and Analyzing Data

We obtained the clinical feature sets and transcriptomic data of HCC samples retrospectively via the database of ICGC (https://dcc.icgc.org/), TCGA (https://portal.gdc.cancer.gov/), and the GEO database (GSE14520 dataset) (https://www.ncbi.nlm.nih.gov/geo/). The TCGA-HCC dataset included 50 normal samples and 374 tumor samples. The ICGC-HCC dataset included 202 normal samples and 243 tumor samples. The GSE14520 dataset included 239 normal samples and 247 tumor samples. Via the GSEA HALLMARK_PI3K_AKT_MTOR_SIGNALING gene set (Supplementary Table 1), we adopted the data of 105 PAGs, the website of which was http://www.broadinstitute.org/gsea/index.jsp.

### 2.4. Extracting the Intersection between DEGs and PAGs

To extract genes (DEGs) that had different expressions in normal samples (*n* = 50) and HCC samples (*n* = 365), we applied the “limma” package. |log2FC| > 2 was conducted as the filter standard for extracting DEGs, on the condition that *p* < 0.05 (otherwise, genes will not make sense). Differentially expressed PAGs were extracted by taking the intersection between the DEGs and PAGs. The results were presented by the volcano, the heatmap, and the boxplot. The “BiocManager,” the “ggpubr,” and the “pheatmap” were incorporated to the adopted R packages. Thereinto, the Venn diagram was accomplished through the website (https://bioinformatics.psb.ugent.be/webtools/Venn/), which does well in taking the intersection between the DEGs and PAGs.

### 2.5. Analysis of the Functional Enrichment of Differently Expressed PAGs

The Kyoto Encyclopedia of Genes and Genomes (KEGG) and Gene Ontology (GO) enrichment pathway analysis, which were proceeded in the Database for Annotation, Visualization, and Integrated Discovery (DAVID) website, analyzed the various physiological functions and pathological processes that were associated with differentially expressed PAGs [21]. Molecular function (MF), cellular component (CC), and biological process (BP) were involved in the GO terms [22]. The results of enrichment analysis were filtered by applying FDR < 0.05, and the realization of visualization of the top ten significant items was achieved by the bubbles and bar diagrams on the application of R 4.0.2 software. The “enrich plot,” the “ggpolt2,” the “DOSE,” and the “Cluster Profiler” were incorporated in installed R packages.

### 2.6. GeneMANIA

GeneMANIA (http://genemania.org) [23] is an online website which can be used to predict the gene function, analyze the gene lists, and conduct the optimal gene function annotation. In this study, the GeneMANIA website was used to investigate the coexpressed genes of differentially expressed PAGs and the underlying interactions between PAGs and their coexpressed genes by using the genomics and proteomics data.

### 2.7. Establishing PAPS

To verify the prognosis-related PAGs, we adopted the multivariate Cox regression analyses as well as univariate Cox regression analyses. The PAPS was established based upon the multivariate Cox regression analysis from TCGA database, followed by validating the prognostic value in the ICGC database and GSE14520 datasets. The equation was explained in the following. The risk score equals to *A* gene expression × R1 + *B* gene expression × R2 + *C* gene expression × R3 + ⋯ (*R* represented the value of regression coefficient which was conducted by multivariate regression analysis). Upon utilization of the “glmnet” package via R software, it carried out the four-gene prognostic signature that was obtained from PAGs. The receiver operating characteristic (ROC) curve was adopted in terms of the evaluation of the constructed prognostic features regarding its predictive prognostic value. Meanwhile, the Kaplan-Meier (K-M) curve was adopted by us for the same purpose as well. We adopted the “rms” package to draw the nomogram so as to predict the patients of their respective survival rates.

### 2.8. The Application of the ONCOMINE Database in This Study

The ONCOMINE database (https://www.oncomine.org/resource/login.html) [24] served as an online website in respect of the tumor-related analyses. Among 44 types of human cancers, CDK1, PITX2, PRKAA2, and SFN expression was analyzed. The gene rank of the top 10% genes, fold change = 2, and *p* = 0.0001 were considered to be threshold parameters. The value of *p* was computed by adopting the results which were given rise by the *t*-test of the students.

### 2.9. cBioPortal Analysis

Several genomics data of human cancers had been identified, analyzed, and visualized in a perspective that was multidimensional by an online website, namely, Cancer Genomics-related cBioPortal (https://www.cbioportal.org/) [25]. The scope of analysis of this website on the alternations of genomic profiles not merely contained the assumed mutations and the alterations of copy numbers but also incorporated deep deletion and amplification. The cBioPortal website was also applied in the calculation regarding the genetic changes in terms of CDK1, PITX2, PRKAA2, and SFN.

### 2.10. The Application of Human Protein Atlas Database in This Study

A human proteome program, namely, the Human Protein Atlas (HPA) database (https://www.proteinatlas.org/) [26], was derived from the Swedish database. HPA functions as an online website regarding the analysis of various human proteins in respect of organs, tissues, and cells by taking the advantage of the technology of proteomics. The technology of proteomics incorporated mass spectrometry-based proteomics, systems biology, transcriptomics, and antibody-based imaging. The data on the protein expression of PITX2 was not included in the database we herein referred to, but the data regarding the expression of CDK1, SFN, and PRKAA2 at protein levels can be accessed via the HPA database.

### 2.11. Analytical Method

The Wilcoxon signed-rank test was adopted in terms of analyzing the genes that had various expressions in respect of the normal tissues and HCC tumor tissues as well. The standardization of the sequencing data of mRNA can be realized by the transformation of log2. The sequencing data of mRNA was adopted via the employment of R 4.0.2 software, and so was the clinical information. At the same time, the Perl languages were adopted to take part in this course. We investigated the enrichment of immune cell infiltration among the samples by means of the CIBERSORT method [27]. The “parallel,” “e1071,” and “BiocManager” were incorporated into the R packages. The correlation between the risk score and the infiltrating immune cells was estimated by a couple of packages (i.e., “limma,” “BiocManager,” “ggtext,” “ggExtra,” “ggpubr,” “ggplot2,” “scales,” and “vioplot”) in R software 4.0.2. The differential half-maximal inhibitory concentration (IC50) of chemotherapeutic drugs between groups of low risk and high risk was computed by the “pRRophetic” package [28]. Sorafenib, Axitinib, Docetaxel, Gefitinib, Cyclopamine, and Dasatinib were the drugs included.

## 3. Results

### 3.1. The Intersection between the DEGs and PAGs

**Figure 1:**
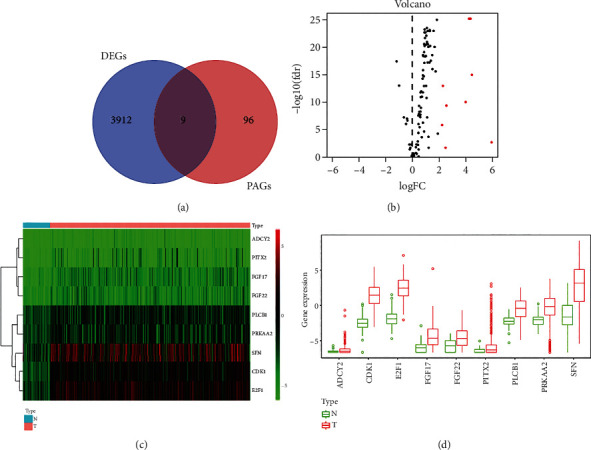
The intersection between the DEGs and PAGs was obtained. (a) 3921 DEGs were identified between normal tissues (|log FC| > 2, FDR < 0.05) and tumor tissues. Nine upregulated PAGs were further identified by taking the intersection between the DEGs and PAGs. (b) Nine upregulated PAGs achieved their visualization by the application of the volcano plot. (c) The heatmap was employed in terms of presenting the nine PAGs of its various expressions. (d) The boxplot was employed in terms of presenting the nine PAGs of its various expressions.

The TCGA was an online platform for the purpose of downloading both the sequencing data of mRNA and homologous clinical information in terms of 50 normal tissue samples as well as 374 HCC tissue samples. M stage, N stage, gender, TNM stage, grade, T stage, and age were what were incorporated regarding the clinicopathological information. Via the HALLMARK_PI3K_AKT_MTOR_SIGNALING gene set of GSEA (Supplementary Table 1), 105 PAGs were obtained. Preceding experiments had indicated that the 105 genes were highly correlated with the PI3K/AKT/mTOR pathway. The Wilcoxon signed-rank test and the “limma” R package identified 3921 DEGs between normal tissues in HCC (|log FC| > 2, FDR < 0.05) and tumor tissues. Nine upregulated PAGs were further identified by taking the intersection between the DEGs and PAGs (Figure 1(a)) and achieved its visualization by the application of the volcano plot (Figure 1(b)). The boxplot (Figure 1(d)) and heatmap (Figure 1(c)) were employed in terms of presenting the nine PAGs of its various expressions as well.

### 3.2. The Results of GO and KEGG Function Enrichment Analyses

**Figure 2:**
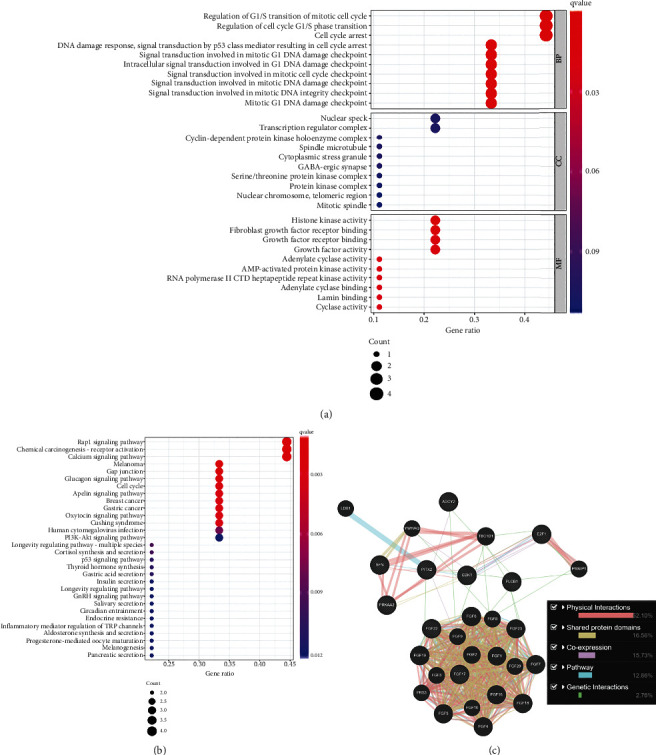
(a) The verification of the biological processes which was associated with nine PAGs was achieved by the GO analyses. (b) The verification of the underlying pathways which was associated with nine PAGs was achieved by the KEGG analyses. (c) The interactions between the nine PAGs and the coexpressed genes were exhibited.

The verification of the biological processes as well as underlying pathways which were associated with nine PAGs was achieved by the GO and KEGG analyses. The most crucial BP was the G1/S transition that regulates the mitotic cell cycle, the most correlated CC was “nuclear speck,” and the most relevant MF was “histone kinase activity” (Figure 2(a)). “Rap1 signaling pathway” was the most crucial signaling pathway (Figure 2(b)). The interactions between the nine PAGs and the coexpressed genes are exhibited in Figure 2(c).

### 3.3. Four Prognostic PAGs Were Identified, and PAPS Was Constructed

**Figure 3:**
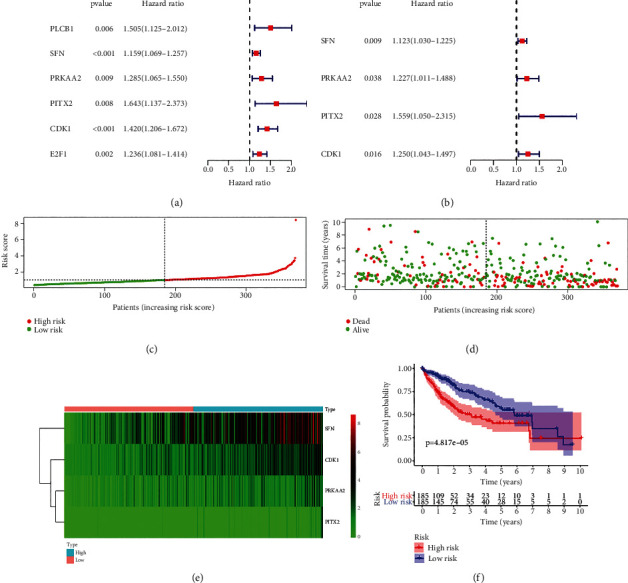
(a) Six prognosis-related PAGs related to the OS of hepatocellular carcinoma patients were determined. (b) CDK1, SFN, PRKAA2, and PITX2 were eventually identified. (c) The PAPS categorized the HCC patients as low risk and high risk based on the median risk score. (d) The scatterplot presented that patients' survival will reduce following the increment of the risk score; the dead patients increased. (e) As was presented in the heatmap, in the low- and high-risk cohort, there existed differential CDK1, PITX2, PRKAA2, and SFN expression. (f) It was observed in the K-M curve that hepatocellular carcinoma patients of low risk had a higher probability of prognosis than high-risk patients.

In identifying the prognostic PAGs, we adopted multivariate Cox regression. In the meantime, we adopted univariate Cox regression analysis as well. The univariate Cox regression analysis determined 6 prognosis-related PAGs correlated with the OS of HCC patients, which were involved in E2F1, PITX2, PRKAA2, PLCB1, SFN, and CDK1 (Figure 3(a)). As was visualized in Figure 3(b), we eventually identified CDK1, SFN, PRKAA2, and PITX2 by conducting multivariate Cox regression analysis. The regression coefficients can be visualized as well (Table 1). Following the expression data of CDK1, PITX2, PRKAA2, and SFN which were chosen and their corresponding regression coefficients, we established PAPS. The equation being applied in this experiment was PAPS = CDK1 expression∗0.222894 + PITX2 expression∗0.443949 + SFN expression∗0.116124 + the expression level of PRKAA2 expression∗0.204509. The above equation was applied in the calculation of the patients regarding their risk scores. Based on the median risk score (Figure 3(c)), the PAPS categorized the HCC patients as low risk and high risk. The scatterplot presented that patients' survival will reduce following the increment of the risk score; the dead patients increased (Figure 3(d)). As was presented in the heatmap (Figure 3(e)), in the low-risk group and the high-risk group, there existed differential CDK1, PITX2, PRKAA2, and SFN expression. It was observed in the K-M curve that HCC patients of high risk had a lower probability of survival than low-risk patients (*p* = 4.817 *E* − 05; Figure 3(f)). There were as many as 185 samples, respectively, in groups of low risk and high risk.

### 3.4. A Higher Prognostic Value of PAPS than That of Various Clinicopathological Features Can Be Obtained

**Figure 4:**
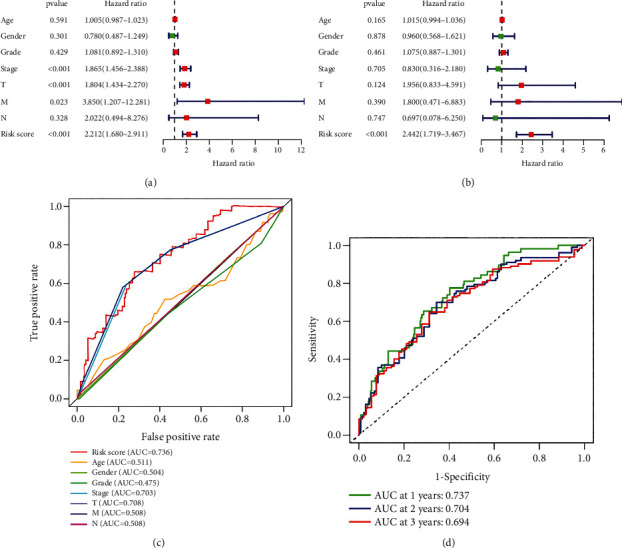
(a) The PAPS had great prognostic value for HCC patients. (b) The PAPS had great independent prognostic value for HCC patients. (c) The constructed PAPS had better prognostic value than various clinicopathological features in HCC. (d) The PAPS had great prognostic value for 1-year, 2-year, and 3-year survival of HCC patients.

In terms of the investigation of the independent prognostic value of PAPS, univariate and multivariate Cox regression analyses were adopted by us in the experiment. In terms of the reliability of our study, we also applied a number of clinicopathological characteristics as interfering factors during the process. The clinicopathologic characteristics incorporated tumor grade, tumor N stage, tumor M stage, tumor T stage, patients' age, and patients' gender. From Figures 4(a) and 4(b), we can conclude that PAPS may be an independent prognostic factor for HCC because *p* < 0.001 and hazard ratio > 1. The ROC curve was applied for the purpose of comparing the prognostic value in various clinicopathological features and in the constructed PAPS. The result of a higher prognostic value of PAPS (AUC = 0.736) than that of various clinicopathological features (AUC ≤ 0.511) (Figure 4(c)) can be obtained from the test. The ROC curve assessed the reliability of PAPS. Meanwhile, the area under the ROC curve played a crucial role statistically; Figure 4(d) presented that the AUC values of 1 year, 2 years, and 3 years were 0.737, 0.704, and 0.694, respectively.

### 3.5. The Upregulation of Four PAGs in HCC

**Figure 5:**
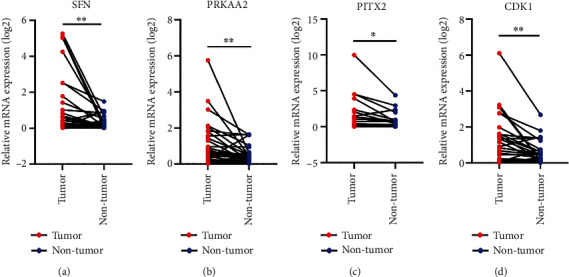
Comparing with the corresponding normal tissue, the qRT-PCR ultimately confirmed the upregulation of (a) SFN, (b) PRKAA2, (c) PITX2, and (d) CDK1 in tumor tissue.

Compared with normal tissue samples through the ONCOMINE database (Supplementary Figure 1A), the expression of mRNA in PRKAA2, SFN, and CDK1 was all promoted in HCC tissue samples. Nevertheless, the data regarding the expression of PITX2 was not incorporated into the ONCOMINE database. The protein levels of PRKAA2, SFN, and CDK1 were upregulated in tumor tissue on the basis of the Human Protein Atlas (HPA) database (Supplementary Figure 1B). Data associated with the expression of PITX2 was not incorporated in HPA. We thereafter applied the cBioPortal online tool to identify the four PAGs of their genetic alternations in HCC. The deep deletion, the amplification, and the missense mutation were all within the observation of the alternation as a result, and the amplification was the most common phenomenon (Supplementary Figure 1C). Compared with the corresponding normal tissue (Figure 5), the qRT-PCR ultimately confirmed the upregulation of SFN, PRKAA2, PITX2, and CDK1 in tumor tissue. The tumor tissue and corresponding normal tissue were collected from 35 pairs of clinical samples.

### 3.6. The Positive Correlation of Clinicopathological Features and PAPS

Further, we explored the association between clinicopathological features and PAPS. The data we adopted consisted of the sequencing data of mRNA and homologous clinical information, and we obtained them via TCGA database. It can be drawn from the experiment that the risk score correlated with the T classification (*p* = 0.00015; Supplementary Figure 2C), the grade (*p* = 1.8*E* − 07; Supplementary Figure 2B), and the stage (*p* = 7.9*E* − 05; Supplementary Figure 2A), and the SFN expression was correlated to the grade (*p* = 0.032; Supplementary Figure 2D). The expression of PRKAA2 was correlated with the T classification (*p* = 0.049; Supplementary Figure 2G), the grade (*p* = 0.00035; Supplementary Figure 2F), and the stage (*p* = 0.022; Supplementary Figure 2E). The expression of PITX2 was correlated with T classification (*p* = 0.00094; Supplementary Figure 2I) and stage (*p* = 0.00011; Supplementary Figure 2H). The expression of CDK1 was also related to the stage (*p* = 0.0034; Supplementary Figure 2J), the grade (*p* = 4.5*E* − 08; Supplementary Figure 2K), and the T classification (*p* = 0.004; Supplementary Figure 2L).

### 3.7. PAPS Presented Great Prognostic Value in GEO (GSE14520) and ICGC Database

**Figure 6:**
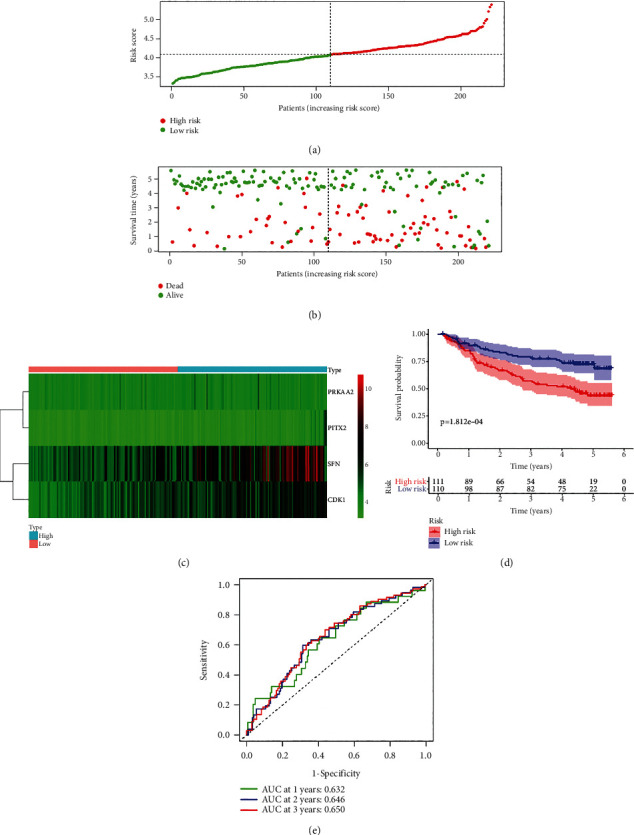
(a) In the test set of the GSE14520 dataset, the HCC patients were classified as low risk and high risk based on the value of the risk score. (b) In the scatterplot, the survival time of patients decreased considerably as the patients' risk scores increased. (c) The evident differential expression of CDK1, PITX2, PRKAA2, and SFN was recognized and presented in the heatmap. (d) A high-risk group had shorter survival than the low-risk group in the K-M curves. (e) The PAPS was better in the predictive significance for 1-year, 2-year and 3-year survival in ROC curves.

**Figure 7:**
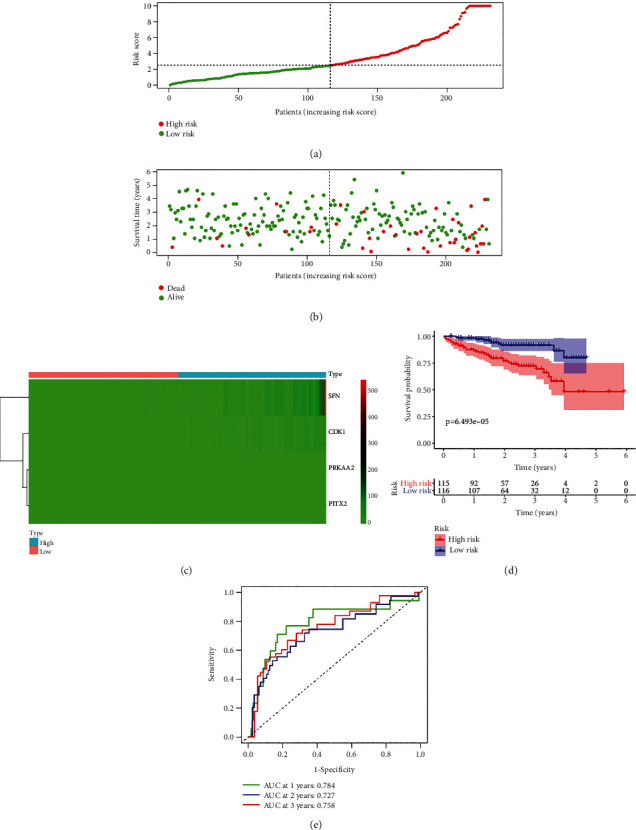
For the betterment of the identification of the prognostic value of PAPS, the data that was derived from the ICGC database was thereafter utilized by us. (a) The HCC patients were divided into groups of low risk and high risk based on the value of the risk score. (b) The survival time of patients has to a large extent decreased when the patients' risk score increased. (c) The evident differential expressions of four PAGs (CDK1, PITX2, PRKAA2, and SFN) in the ICGC database were recognized as well as being presented by heatmap. (d) In the K-M curve, the survival probability of patients with low risk was higher than that with high risk. (e) The ROC curve showed that this signature was more predictive of 1-year, 2-year, and 3-year survival.

**Figure 8:**
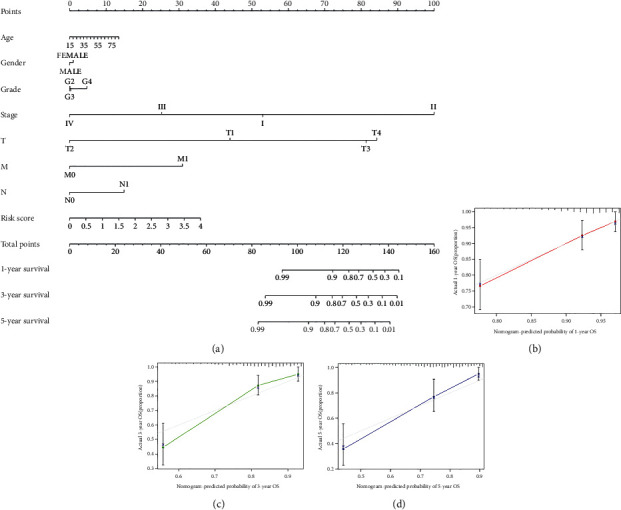
(a) The total point showed the survival prediction of HCC patients in 1-year survival, 3-year survival, and 5-year survival. (b–d) Good consistency between the realistic outcome (1-year, 3-year, and 5-year) and the predictive survival in TCGA database was revealed by the calibration chart of PAPS.

We thereafter downloaded GEO (GSE14520) and ICGC database in terms of the verification of the effectiveness and the accuracy of PAPS. In the test set of the GSE14520 dataset, we classified the HCC patients as low risk and high risk based on the value of the risk score shown in Figure 6(a). In the scatterplot (Figure 6(b)), the survival time of patients decreased considerably as the patients' risk scores increased. The heatmap (Figure 6(c)) recognized and presented the evident differential expressions of CDK1, PITX2, PRKAA2, and SFN. The K-M curves revealed that a high-risk group had shorter survival than the low-risk group (*p* = 1.812*E* − 04; Figure 6(d)). The ROC curves revealed that PAPS was better in the predictive significance for 1-year, 2-year, and 3-year survival (1-year AUC equaled 0.632, 2-year AUC equaled 0.646, and 3-year AUC equaled 0.650; Figure 6(e)). For the betterment of the identification of the prognostic value of PAPS, the data that was derived from the ICGC database was thereafter utilized by us. We also divided the HCC patients into groups of low risk and high risk based on the value of the risk score (Figure 7(a)). Likewise, the survival time of patients has to a large extent decreased when the patients' risk score increased (Figure 7(b)). The evident differential expressions of 4 PAGs (CDK1, PITX2, PRKAA2, and SFN) in the ICGC database (Figure 7(c)) were recognized as well as being presented by the heatmap. In the K-M curve, the survival probability of patients with low risk was higher than that with high risk (*p* = 6.493*E* − 05; Figure 7(d)). The ROC curve also showed that this feature was more predictive of 1-year, 2-year, and 3-year survival (1-year AUC equaled 0.784, 2-year AUC equaled 0.727, and 3-year AUC equaled 0.758; Figure 7(e)). Furthermore, the predictive nomogram was constructed for HCC patients. The nomogram was established upon PAPS and various clinicopathological features, which could estimate the survival risk in monitoring HCC patients. The survival of HCC patients in 1 year, 3 years, and 5 years was all in the prediction of the total point (Figure 8(a)). Meanwhile, a good consistency between the realistic outcome (1 year, 3 years, and 5 years) and the predictive survival in TCGA database (Figures 8(b)–8(d)) was revealed by the calibration chart of PAPS.

### 3.8. The PAPS Was Positively Correlated with Immune Cell Infiltration and the Expression Levels of Immune Checkpoints

**Figure 9:**
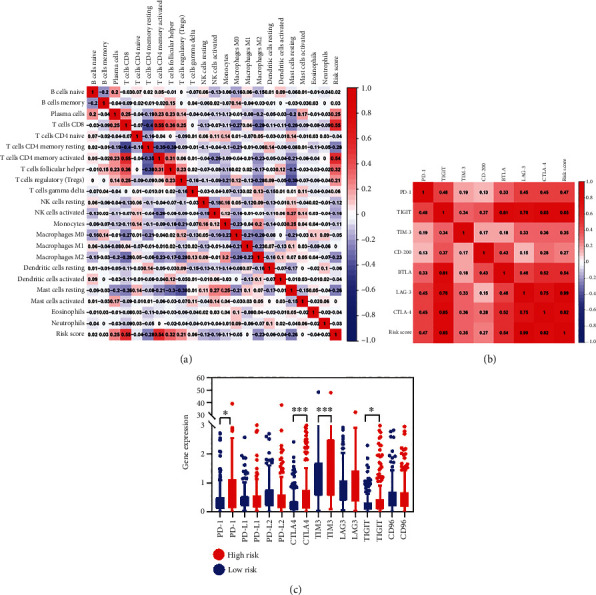
(a) The correlation over the relative proportion of infiltrating immune cells and PAPS in HCC was presented. (b) The correlation between the PAPS and a couple of immune checkpoint expression (TIGIT, PD-L1, CD96, LAG3, PD-L2, TIM3, CTLA4, and PD-1) was presented. (c) Compared to that of the low-risk group, CTLA-4, TIM-3, TIGIT, and PD-1 had a more evident expression in groups of high risk.

In terms of exploring the predictive roles of PAPS in TME, the concrete constituent of infiltrating immune cells in HCC swatches was calculated with the CIBERSORT method. Subsequently, we conducted a correlation analysis of the relative proportion of infiltrating immune cells and PAPS in HCC. The results indicated that the risk score of PAPS was significantly positively correlated with the relative proportion of CD8+ T cells (cor = 0.55), CD4 activated memory T cells (cor = 0.54), and the follicular helper T cells (cor = 0.32) (Figure 9(a)). The inefficiency regarding the functioning of immune checkpoint inhibitors in some of the HCC patients was revealed by recent studies [29], but the reason for it had not been fully explained. Therefore, the investigation of the correlation between the PAPS and a couple of immune checkpoint expression (TIGIT, PD-L1, CD96, LAG3, PD-L2, TIM3, CTLA4, PD-1) was conducted by us. And we have exhibited the results in Figure 9(b). The results indicated that there existed a positive association between the risk score of PAPS and the expression of PD-1 (cor = 0.47), TIGIT (cor = 0.85), TIM-3 (cor = 0.35), BTLA (cor = 0.54), LAG-3 (cor = 0.99), and CTLA-4 (cor = 0.82). Compared to that in the low-risk group (Figure 9(c)), CTLA-4, TIM-3, TIGIT, and PD-1 had a more evident expression in groups of high risk. It revealed that PAPS could serve as a reflection regarding the above immune checkpoint expression. The predictive ability of PAPS was immense regarding immune checkpoint inhibitors of their sensitivity in HCC.

### 3.9. The PAPS Presented the Differential Drug Sensitivity between the Groups of Low Risk and High Risk

**Figure 10:**
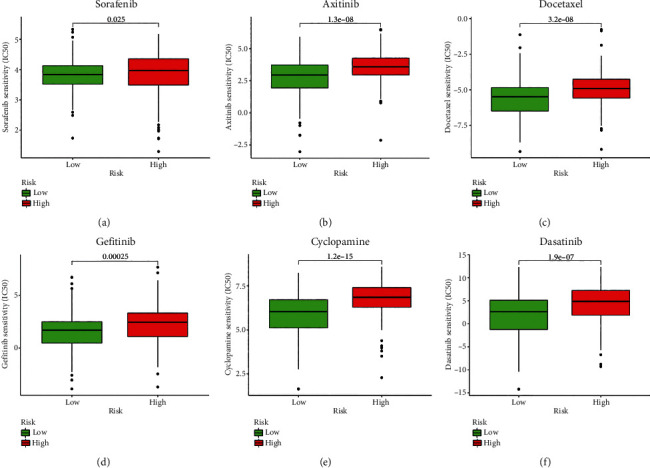
The PAPS presented the differential drug sensitivity between the group of low risk and high risk. (a) The group of low risk obtained a lower IC50 value of sorafenib. (b) The group of low risk obtained a lower IC50 value of Axitinib. (c) The group of low risk obtained a lower IC50 value of Docetaxel. (d) The group of low risk obtained a lower IC50 value of Gefitinib. (e) The group of low risk obtained a lower IC50 value of Cyclopamine. (f) The group of low risk obtained a lower IC50 value of Dasatinib.

By analyzing the association between the drug sensitivity and risk score, we aimed to explore the clinical applications of PAPS. The experiment indicated that the group of low risk obtained a lower IC50 value of sorafenib (*p* = 0.025; Figure 10(a)), Axitinib (*p* = 1.3*E* − 08; Figure 10(b)), Docetaxel (*p* = 3.2*E* − 08; Figure 10(c)), Gefitinib (*p* = 0.00025; Figure 10(d)), Cyclopamine (*p* = 1.2*E* − 15; Figure 10(e)), and Dasatinib (*p* = 1.9*E* − 07; Figure 10(f)). As shown in Figure 10, the lower IC50 values represented the higher drug sensitivity.

## 4. Discussion

Though a giant leap had been made, respectively, upon the progress of prognosis prediction, diagnosis, and treatment strategies of HCC, HCC still accounted for the leading cause of cancer death all over the world [30]. The heterogeneous malignancy was characterized by possessing complicated molecular subtypes with genetic and genomic alterations [31]. Especially the PI3K/AKT/mTOR pathway that performs an essential effect on the tumorigenesis of HCC including proliferation, metastasis, and resistance to chemotherapy, any alterations of the PI3K/AKT/mTOR pathway could cause pathological processes of hepatocellular carcinoma [32]. In clinical trials of HCC, the inhibitory agents of the PI3K/AKT/mTOR pathway were completely investigated and assessed [33]. For example, sorafenib had better therapeutic efficacy in combination with PI3K inhibitors (PI-103) by targeting the PI3K/AKT/mTOR pathway [34]. However, only a part of patients can benefit from the inhibitory agents because of different molecular profiles [33]. Therefore, it is reasonable to identify a prognostic signature based on PAGs and validate its predictive performance for predicting chemotherapy response in HCC.

The current study showed that it is crucial to set up an early predictive model for prognosis when it comes to human cancer [35, 36], and various therapeutic strategies are supposed to be established in line with the diverse prognosis of patients. At the same time, an emerging trend indicated that the researchers would be willing to identify molecular signatures for predicting prognosis, which was constructed by integrating genomics with bioinformatics [37]. Therefore, we attempted to establish a powerful model for prognosis prediction and chemotherapy response evaluation by using bioinformatic methods, which can provide precious directions for guiding clinical intervention. The PAPS was developed by utilizing the transcriptome data and clinicopathological information from TCGA-HCC specimens, followed by conducting the verification of the prognostic value in ICGC-HCC specimens and GSE14520-HCC specimens as well. Nine differently expressed PAGs were determined, which were correlated with various biological processes and signaling pathways. In particular, the PI3K/AKT pathway was involved in them. Subsequently, we employed the Cox regression analysis to identify four prognosis-related PAGs (CDK1, PITX2, PRKAA2, and SFN), which were utilized to develop PAPS. The upregulation of CDK1, PITX2, PRKAA2, and SFN was confirmed in our clinical samples.

Emerging evidence determined that TME was pivotal for cancer development and progression [38, 39]. Giannone et al. proposed that hyperactivation of the PI3K/AKT pathway could induce the immune tolerance in TME, thereby lowering the chemotherapy response of immune checkpoint inhibitors (ICIs) [17]. Jiang et al. mentioned that the hyperactivation of the PI3K/AKT pathway was correlated with the immune microenvironment and the response to chemotherapy in tumor cells [40]. HCC, known as a malignant tumor, was always with cirrhosis and chronic liver inflammation. And gene markers' expression in some HCC cases indicated the presence of an immune response, which provides new insights into the immunotherapy of HCC [41]. However, a large quantity of HCC patients presented a relatively low response to immunotherapy [42]. The prognosis of HCC patients was widely recognized to be correlated with activated innate immune as well as the inflammatory gene expression of TILs [43, 44]. Therefore, we further employed the CIBERSORT algorithm to calculate the relative ratio of TILs in HCC samples, and we evaluated the relation of the PAPS and the proportion of TILs in HCC. TILs were correlated with HCC prognosis and potent to be immunotherapeutic targets in HCC [45]. The present study demonstrated that PAPS was correlated with the relative proportion of follicular helper T cells, CD4 activated memory T cells, and CD8 T cells. Liu et al. mentioned that the HCC patients with PD-1+ TIGIT+ type of CD8+ T cells were related to rapid cancer progression and poor prognosis [46]. The exhaustion of Tfh cells was caused by the upregulation of PD-1 in HCC, which was correlated with the progressive tumor stage [47]. The reduced Tfh cells had the potential to be a therapeutic target as well as a prognostic biomarker in HCC [48]. It follows that the PAPS has the potential to predict immune cell infiltration as well as prognosis evaluation. A significant advance in immunotherapy of HCC had been achieved, especially applying ICIs may become optional therapeutics [49]. Increasing evidence indicated that targeting PD-1/CTLA-4 may recover antitumor immunity [50]. Targeted therapeutics targeting PD-L1/PD1 (nivolumab and pembrolizumab) may be a promising subsequent-line therapy in HCC, but the efficacy of the above agents was relatively low [51]. T cell immunoglobulin mucin-3 (TIM-3) was hailed as a new immune checkpoint and had the potential to direct the prognosis; it even presented a great therapeutic effect against tumors in HCC [52]. TIGIT was also involved in the immune surveillance in TME and had an antitumor effect [53], and it also induced immunosuppression towards CD8 T cells [54]. The upregulation of LAG-3 was detected in infiltrating CD8+ T cells, and it was even reported to inhibit T cell response in HCC [55]. Therefore, we established the PAPS to evaluate the reactivity of immune checkpoint inhibitors. Our research indicated that the group of high risk obtained higher scores of TIM-3, PD-1, CTLA-4, and TIGIT, suggesting that PAPS could distinguish the HCC patients who had a higher response to immune checkpoint inhibitors. The great prediction efficiency of PAPS applied to predict the response to immune checkpoint inhibitors provided a foundation for clinical immunotherapeutic decision-making. Hopefully, the novel PAPS has the potential to promote the process of HCC individualized treatment.

The nomogram composed of PAPS and various clinicopathological characteristics was further established and validated, which is aimed at improving the predictive accuracy of PAPS. For example, Ying et al. had developed a nomogram composed of inflammatory response biomarkers, which had a great ability to enhance the predictive power for colorectal cancer patients suffering surgical resection [56]. In general, the novel prediction method had great potentialities in enhancing predictive accuracy as well as predicting survival for individual patients [57].

Our study indicated that PAPS can also effectively suggest the sensitivity of several chemotherapeutic drugs in HCC. Sorafenib was regarded as the first-line drug for HCC chemotherapy, and it was under the approval of the Food and Drug Administration (FDA) [58]. However, the resistance of sorafenib to the drug still existed, and the PI3K/AKT pathway was considered the underlying mechanism of resistance to sorafenib [59]. Our study demonstrated that PAPS was composed of four PAGs, and PAPS can reveal the drug sensitivity to sorafenib in HCC. Some research indicated that Axitinib was considered the second-line drug for advanced HCC, and it had moderate activity and tolerable toxicity for HCC patients with the failure of sorafenib monotherapy [60]. In pancreatic adenocarcinoma, the drug resistance to Axitinib can be reversed by applying the PI3K inhibitor [61]. Our research presented great predictive performance of PAPS in predicting the drug sensitivity to Axitinib in HCC; it suggested that the PI3K/AKT/mTOR pathway might be involved in the mechanism of Axitinib resistance in HCC, but the concrete mechanism should be further explored. Interestingly, Docetaxel induced HCC cell apoptosis and inhibited the PI3K/AKT signaling pathway [62]; it was consistent with our results showing that PAPS can exhibit the drug sensitivity of Docetaxel in HCC. Gefitinib had a great therapeutic effect as the epidermal growth factor receptor (EGFR) inhibitor; it was capable of restraining the proliferation of HCC cells and triggering the apoptosis of the cell by combining with genistein as well [63]. In particular, its therapeutic effects can be strengthened in combination with blocking the PI3K/AKT/mTOR signaling pathway in triple-negative breast cancers [64]. It was in accordance with our results showing PAPS predicted the drug sensitivity of Gefitinib well in HCC. Cyclopamine, the inhibitor of the sonic hedgehog (Shh) signaling pathway, may regulate cell apoptosis by downregulating Bcl-2 expression [65]. The radiosensitivity of HCC patients may enhance in combination with the Shh inhibitor [66]. Dormoy et al. had put forward that Cyclopamine also suppressed the activation of the PI3K/AKT pathway in renal cell carcinoma [67]. The present study showed that PAPS can imply the drug sensitivity of Cyclopamine in HCC. However, whether the PI3K/AKT pathway can be inhibited by Cyclopamine in HCC needs to be further explored. Dasatinib was reported to enhance the antitumor effect of irinotecan in HCC, and it provided HCC patients with a neoteric insight in respect of the new strategy [68]; the chemotherapy response to Dasatinib can also be revealed by PAPS in our study.

Our research has several strengths. Firstly, the predictive performance of PAPS was confirmed in multiple datasets, and PAPS showed great reliability and robustness in different datasets. Secondly, a comprehensive analysis of the correlation of the PAPS with immune infiltration and chemotherapy response was conducted. What is more, the expression of PITX2, PRKAA2, SFN, and CDK1 was validated in clinical samples via qRT-PCR technology. However, our study also had some limitations, the concrete carcinogenic mechanism of PITX2, PRKAA2, SFN, and CDK1 in HCC should be further explored in cell and animal experiments, and the expression levels of PITX2, SFN, CDK1, and PRKAA2 should be verified in larger size of clinical HCC samples. Western blot and immunohistochemistry technologies could be involved in further study.

## 5. Conclusions

For the purpose of verifying PAGs' prognostic value as well as figuring out the correlation between PAGs and the corresponding immune infiltration in HCC, a new PI3K/AKT/mTOR pathway-associated prognostic signature (PAPS) was established by us with integrated bioinformatic methods. It presented immense predictive performances in the prognosis of HCC patients. Meanwhile, the nomogram composed of PAPS and various clinicopathological characteristics enhanced the predictive accuracy of PAPS. We trained the prognostic value of PAPS via TCGA database and thereafter had it tested via the GEO database (GSE14520 dataset) and ICGC database. A conclusion can be drawn that compared with the other clinicopathological characteristics, there is a better prognostic value in PAPS, and PAPS was also correlated with immune microenvironment and chemotherapy response in HCC. The PAPS may hopefully present the immunotherapeutic efficacy and function as a valuable tool to direct the precise treatment for HCC patients.

## Figures and Tables

**Table 1:** Four PAGs (CDK1, PITX2, PRKAA2, and SFN) were eventually identified by conducting a multivariate Cox regression analysis in HCC.

Gene id	Coefficient	HR	HR.95L	HR.95H	*p* value
SFN	0.116124	1.123135	1.029512	1.225273	0.008925
PRKAA2	0.204509	1.226922	1.011449	1.488299	0.037941
PITX2	0.443949	1.558850	1.049609	2.315162	0.027815
CDK1	0.222894	1.249688	1.043175	1.497084	0.015579

Note: PAGs: phosphatidylinositol 3-kinases (PI3Ks)/protein kinase B (AKT)/mammalian target of rapamycin (mTOR)-associated genes; HCC: hepatocellular carcinoma; HR: hazard ratio.

## Data Availability

All the data involved in this study were obtained from the GEO website (https://www.ncbi.nlm.nih.gov/geo/), TCGA website (https://portal.gdc.cancer.gov/), and ICGC website (https://dcc.icgc.org/).

## Supplementary Materials

Supplementary 1 Supplementary Figure 1: (A) compared with normal tissue samples through the ONCOMINE database, the expression of mRNA in PRKAA2, SFN, and CDK1 was all promoted in HCC tissue samples. Nevertheless, the data regarding the expression of PITX2 was not incorporated into the ONCOMINE database. (B) The protein levels of PRKAA2, SFN, and CDK1 were upregulated in tumor tissue on the basis of the Human Protein Atlas (HPA) database. Data associated with the expression of PITX2 was not incorporated in HPA. (C) We thereafter applied the cBioPortal online tool to identify the four PAGs of their genetic alternations in HCC.

Supplementary 2 Supplementary Figure 2: the association of clinicopathological features and PAPS (risk score). (A) The PAPS correlated with the tumor stage. (B) The PAPS correlated with the tumor grade. (C) The PAPS correlated with the tumor T classification. (D) The expression of SFN was associated with the tumor grade. (E) The expression of PRKAA2 was correlated with the tumor stage. (F) The expression of PRKAA2 was correlated with the tumor grade. (G) The expression of PRKAA2 was correlated with the tumor T classification. (H) The expression of PITX2 was correlated with the tumor stage. (I) The expression of PITX2 was correlated with tumor T classification. (J) The CDK1 expression was also correlated with the tumor stage. (K) The CDK1 expression was correlated with tumor grade. (L) The CDK1 expression was correlated with tumor T classification.

Supplementary 3 Supplementary Table 1: the gene list of 105 PAGs downloaded from the GSEA online website.
