# Supplementary figures and images for: PI3K/AKT/mTOR Pathway-Associated Genes Reveal a Putative Prognostic Signature Correlated with Immune Infiltration in Hepatocellular Carcinoma
Source: Dis Markers. 2022 May 9;2022:7545666. doi: 10.1155/2022/7545666 (PMC9112180; doi:10.1155/2022/7545666)

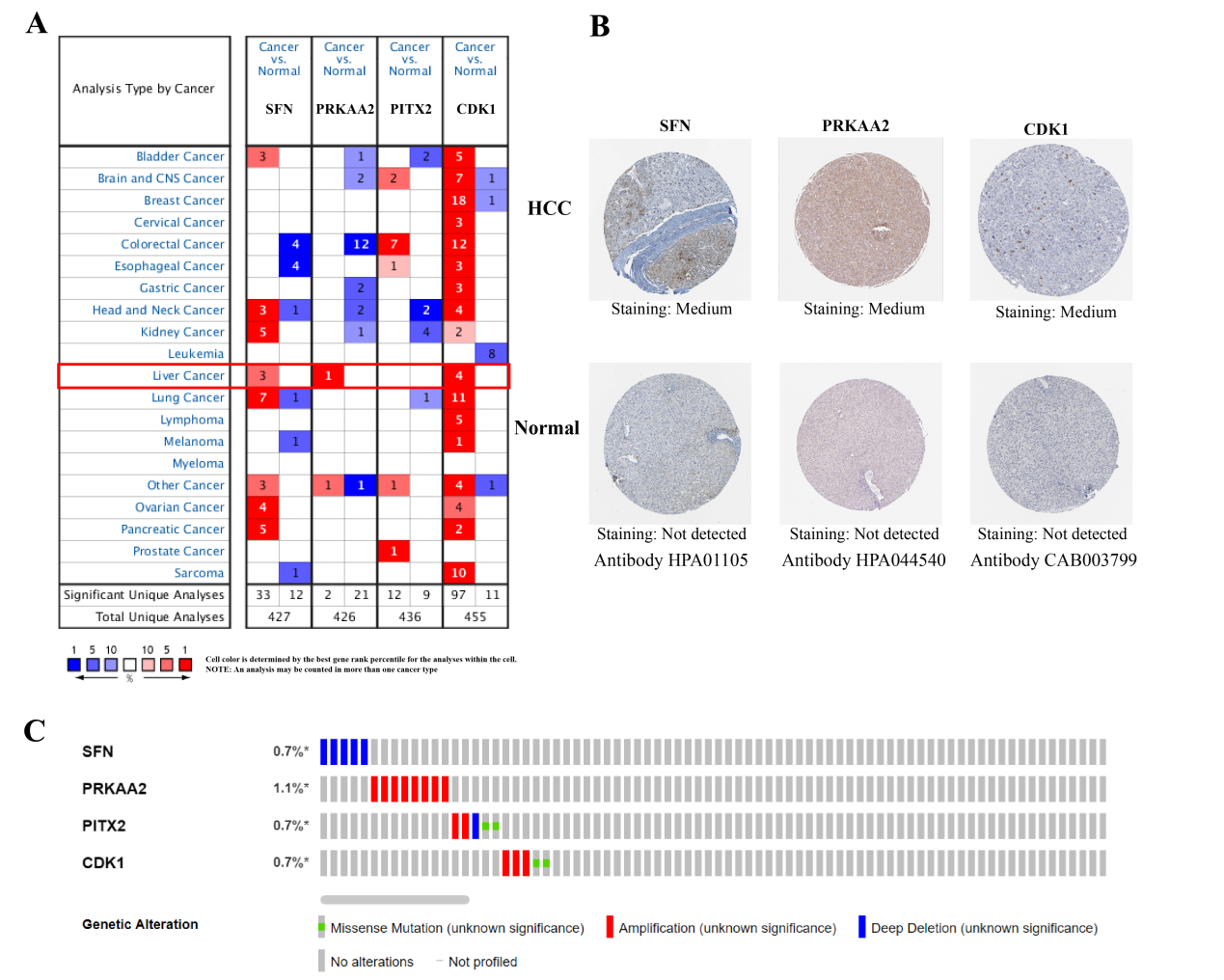

Supplement: Supplementary 1 — Supplementary Figure 1: (A) compared with normal tissue samples through the ONCOMINE database, the expression of mRNA in PRKAA2, SFN, and CDK1 was all promoted in HCC tissue samples. Nevertheless, the data regarding the expression of PITX2 was not incorporated into the ONCOMINE database. (B) The protein levels of PRKAA2, SFN, and CDK1 were upregulated in tumor tissue on the basis of the Human Protein Atlas (HPA) database. Data associated with the expression of PITX2 was not incorporated in HPA. (C) We thereafter applied the cBioPortal online tool to identify the four PAGs of their genetic alternations in HCC. [file 7545666.f1.png]

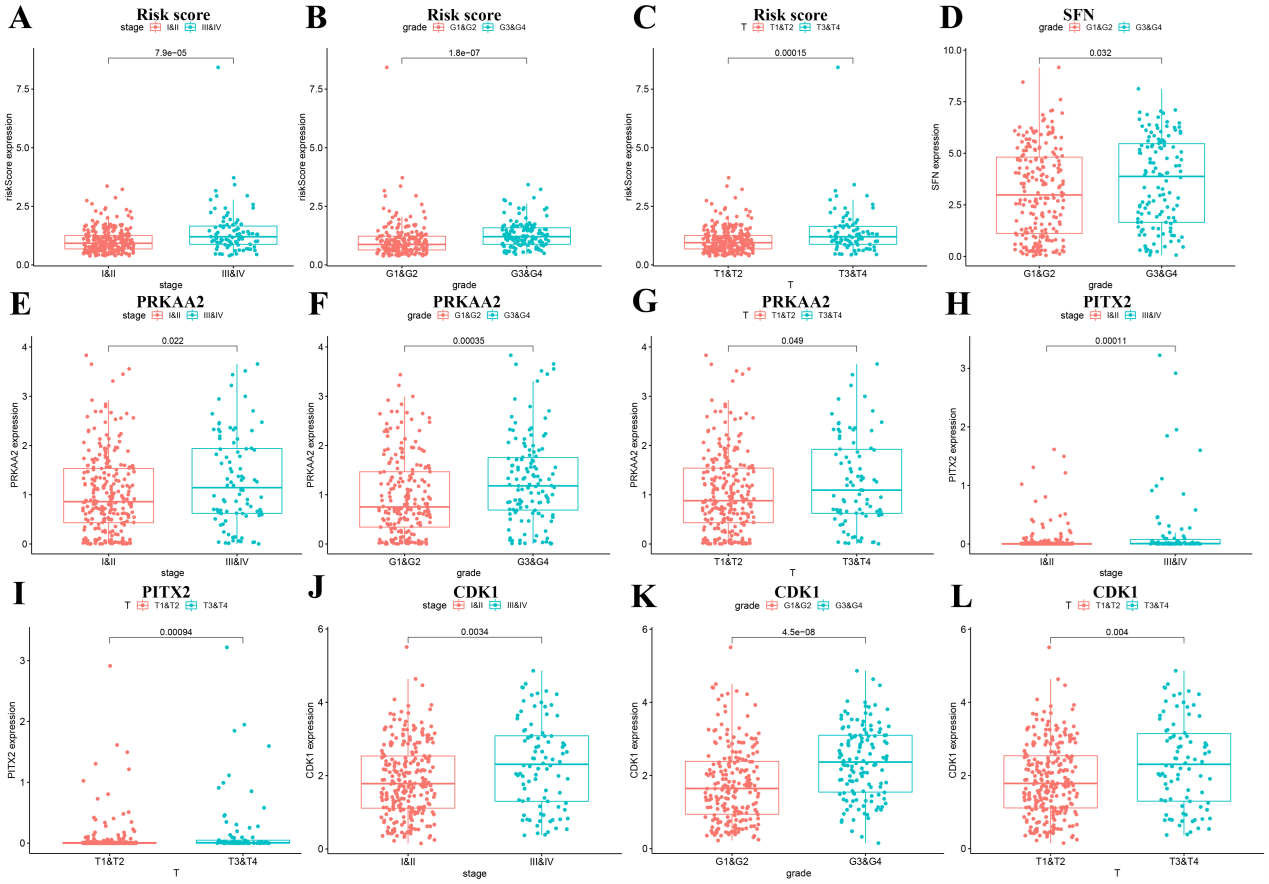

Supplement: Supplementary 2 — Supplementary Figure 2: the association of clinicopathological features and PAPS (risk score). (A) The PAPS correlated with the tumor stage. (B) The PAPS correlated with the tumor grade. (C) The PAPS correlated with the tumor T classification. (D) The expression of SFN was associated with the tumor grade. (E) The expression of PRKAA2 was correlated with the tumor stage. (F) The expression of PRKAA2 was correlated with the tumor grade. (G) The expression of PRKAA2 was correlated with the tumor T classification. (H) The expression of PITX2 was correlated with the tumor stage. (I) The expression of PITX2 was correlated with tumor T classification. (J) The CDK1 expression was also correlated with the tumor stage. (K) The CDK1 expression was correlated with tumor grade. (L) The CDK1 expression was correlated with tumor T classification. [file 7545666.f2.png]
