# Supplementary material for: PI3K/AKT/mTOR Pathway-Associated Genes Reveal a Putative Prognostic Signature Correlated with Immune Infiltration in Hepatocellular Carcinoma
Source: Dis Markers. 2022 May 9;2022:7545666. doi: 10.1155/2022/7545666 (PMC9112180; doi:10.1155/2022/7545666)
Supplement: Supplementary 3 — Supplementary Table 1: the gene list of 105 PAGs downloaded from the GSEA online website. [file 7545666.f3.docx]

**Supplementary Table 1:** The gene list of 105 PAGs downloaded from the GSEA online website.

| [HALLMARK_PI3K_AKT_MTOR_SIGNALING](https://www.gsea-msigdb.org/gsea/msigdb/cards/HALLMARK_PI3K_AKT_MTOR_SIGNALING.html) | | | | |
| --- | --- | --- | --- | --- |
| ACACA | CLTC | IL2RG | PDK1 | RIT1 |
| ACTR2 | CSNK2B | IL4 | PFN1 | RPS6KA1 |
| ACTR3 | CXCR4 | IRAK4 | PIK3R3 | RPS6KA3 |
| ADCY2 | DAPP1 | ITPR2 | PIKFYVE | RPTOR |
| AKT1 | DDIT3 | LCK | PIN1 | SFN |
| AKT1S1 | DUSP3 | MAP2K3 | PITX2 | SLA |
| AP2M1 | E2F1 | MAP2K6 | PLA2G12A | SLC2A1 |
| ARF1 | ECSIT | MAP3K7 | PLCB1 | SMAD2 |
| ARHGDIA | EGFR | MAPK1 | PLCG1 | SQSTM1 |
| ARPC3 | EIF4E | MAPK10 | PPP1CA | STAT2 |
| ATF1 | FASLG | MAPK8 | PPP2R1B | TBK1 |
| CAB39 | FGF17 | MAPK9 | PRKAA2 | THEM4 |
| CAB39L | FGF22 | MAPKAP1 | PRKAG1 | TIAM1 |
| CALR | FGF6 | MKNK1 | PRKAR2A | TNFRSF1A |
| CAMK4 | GNA14 | MKNK2 | PRKCB | TRAF2 |
| CDK1 | GNGT1 | MYD88 | PTEN | TRIB3 |
| CDK2 | GRB2 | NCK1 | PTPN11 | TSC2 |
| CDK4 | GRK2 | NFKBIB | RAC1 | UBE2D3 |
| CDKN1A | GSK3B | NGF | RAF1 | UBE2N |
| CDKN1B | HRAS | NOD1 | RALB | VAV3 |
| CFL1 | HSP90B1 | PAK4 | RIPK1 | YWHAB |
| GSEA: gene set enrichment analysis; PAGs: PI3K/AKT/mTOR pathway-associated genes | | | | |
